# Don’t Make the Best of It, Make It Better: Matching to Residency Programs During COVID-19

**DOI:** 10.1017/cjn.2020.236

**Published:** 2021-01

**Authors:** Fraser Moore, Sarah Bouhadoun, Asli Buyukkurt, Stuart Lubarsky

**Affiliations:** Department of Neurology, Institute for Health Sciences Education, McGill University, Montréal, Canada; McGill University Medical Student (Med-4), Montréal, Canada; McGill University Neurology Resident (PGY-2), Montréal, Canada; Department of Neurology, Institute for Health Sciences Education, McGill University, Montréal, Canada

**Keywords:** medical students, neurology, residency

The COVID-19 pandemic has had a profound effect on all aspects of health care as well as medical education. In the Canadian context, the Association of Faculties of Medicine of Canada (AFMC) announced in May that all interviews of medical students applying for 2021 Canadian Resident Matching Service (CaRMS) positions will be in a virtual format. At the AFMC board meeting on June 18, it was decided that there will be no visiting electives for the medical students in the Class of 2021 for the duration of the 2020–2021 academic year.^1^

Medical students use both visiting electives and interviews to gather information about different residency programs and to demonstrate their interest in particular residency programs. Students use these visits to meet program directors and faculty members, get to know residents, and visit the program training sites.^2^ As a result of the changes made in response to the COVID-19 pandemic, the class of 2021 will not have the same opportunity to gather this knowledge about residency programs. Students already report that the residency application process is frustrating and too subjective,^3^ and the pandemic-related changes and uncertainty could make this worse.^4^

Students also use electives and interviews as a unique opportunity to experience the city itself, independently of the academic program at a given university. This may be of particular importance in a nation the size of Canada. CaRMS data have shown that the number one most important factor for program location choice is the “impression of the town/city.”^5^ The COVID-19 pandemic has made it impossible to visit cities during electives and interviews and very difficult at other times.

How important is it for a medical student to visit residency programs in person? In 2020, approximately 55% of Canadian medical graduates who matched to a residency program in Canada had previously completed an elective in that discipline at the school to which they matched.^6^ For students who matched to Neurology, it was 79%. The published literature on selection of residency programs provides further support to the idea that in-person contact with a residency program is a critical factor in medical students’ decision-making.^2,7,8^

How can medical students in the class of 2021 learn about and evaluate the strengths of different residency programs? The article by Mirian and colleagues in this issue of the Journal^9^ provides advice to medical students applying to adult and pediatric neurology programs. Rather than asking factual questions about research or elective opportunities, the answers to which are often readily available on the CaRMS or program websites, Mirian and colleagues make a strong case for students to develop an “actionable plan” for their evaluation of residency programs. They encourage students to ask residency programs more in-depth questions about the structure and culture of the program. These include how the program’s selection committee will address inequalities in the selection process (such as the advantage that students from a “home school” may have); the adaptability of the program in terms of clinical structure, wellness, safety, and research opportunities in the pandemic era; and the availability of guidance in the form of mentorship and career planning.

Mirian and colleagues also provide advice to residency programs about how to inform students about their program in the absence of in-person electives and interviews. Strategies include improving program websites, allowing applicants to attend virtual academic half-days, hosting virtual meetings with the program director or residents, posting informative videos, hosting virtual tours or information sessions, and connecting applicants with existing residents. Other authors have echoed these suggestions^4,10^ and included additional suggestions such as virtual social gatherings, an enhanced social media presence, testimonials from former residents, and resident-led blogs.^2,7,11^ Importantly, these strategies are not only a means to inform students but they also provide opportunities for programs to showcase their strengths and recruit students.

Mirian and colleagues suggest that programs which successfully adapt their educational strategies to COVID-19 “likely have a well-established foundation in clinical and teaching excellence.”^9^ However, one could argue that the opposite may be true. While the response of a program to a stressor can reflect where it started, it can also illustrate where it is planning to go. Ideally, all programs should be making continuous efforts toward improvement, and the current pandemic provides an opportunity for all programs to innovate.

The article by Mirian and colleagues does not address how residency programs can best gather information about students in the absence of visiting electives and in-person interviews. They suggest that applicants should find answers to fact-based questions about the program on the CaRMS or program websites and focus their virtual face time with the program on more specific details. Perhaps a similar suggestion could be made to the programs. For instance, the answer to traditional interview questions such as “why do you want to be a neurologist” can often be found in the applicant’s written personal statement. With less in-person time with medical students available to residency programs, focusing on interview questions that could help get to know the applicant as a person, team member, and learner could be a more effective use of time.

Mirian and colleagues also do not address the process that residency programs use to select residents. The CaRMS application process has been criticized for its bias and lack of objectivity.^3^ The COVID-19 pandemic may offer residency programs the opportunity to critically review this selection process,^12^ increase objectivity, and reduce the emphasis on perceived “fit”^13^ and familiarity with the program.^12,14^ The use of a defined set of criteria to select applicants for interviews,^13^ more structured interview formats,^12^ and greater transparency about the selection process used^3^ are potential strategies for improvement.

There is no question that the COVID-19 pandemic has provided many challenges for medical education. However, as Mirian and colleagues have shown in their thoughtful article, the pandemic is also an opportunity for both medical students and residency programs to improve the residency selection process.
